# Pressure dependent characteristics of spinels AIn_2_S_4_ (A = Fe, Ni) for spintronic applications: mechanical stable, thermodynamic and spin-polarized electronic behavior

**DOI:** 10.1039/d6ra00659k

**Published:** 2026-07-06

**Authors:** M. Aslam Khan, Shanawer Niaz, N. A. Noor, Sohail Mumtaz, Rabia Iqbal, Hosam O. Elansary

**Affiliations:** a Institute of Physics, Khwaja Fareed University of Engineering and Information Technology Rahim Yar Khan 64200 Pakistan; b Department of Physics, University of Sargodha 40100 Sargodha Pakistan naveedcssp@gmail.com; c Department of Chemical and Biological Engineering, Gachon University 1342 Seongnamdaero, Sujeong-gu Seongnam-si 13120 Republic of Korea sohail.ahmed2015@gmail.com; d School of Materials Science and Engineering, Zhengzhou university Henan China; e Prince Sultan Bin Abdulaziz International Prize for Water Chair, Prince Sultan Institute for Environmental, Water and Desert Research, King Saud University Riyadh Saudi Arabia

## Abstract

Spintronics is an emerging route for next-generation electronics that exploits spin-polarized materials, typically realized in semiconductors and metal through intrinsic magnetic orderings. In current work, mechanical, structural, thermodynamic, and electronic properties for AIn_2_S_4_ (A = Fe, Ni) spinels are examined in detail utilizing density functional theory (DFT) calculations. Optimized lattice parameters are 10.53 Å for FeIn_2_S_4_ and 10.43 Å for NiIn_2_S_4_, in close agreement with reported experimental values. The formation enthalpies of −0.91 eV for FeIn_2_S_4_ and −0.80 eV for NiIn_2_S_4_ indicate that both compounds are thermodynamically stable, with the FeIn_2_S_4_ phase being comparatively more stable. The elasticity demonstrates mechanical stability and ductility, as shown by Poisson's ratios of 0.29 and 0.30 and *B*_0_/*G* values of 2.05 and 2.16 for FeIn_2_S_4_ and NiIn_2_S_4_, respectively. Spin-polarized band structures and density of states (DOS) represent half-metallic nature and the bandgap progressively increase as pressure increases from 0 to 4 GPa for spin up channel for both spinels. The computed total magnetic moment of 4*µ*_B_ f.u.^−1^ for FeIn_2_S_4_ and 2*µ*_B_ f.u.^−1^ for NiIn_2_S_4_ mainly stems from Fe/Ni ions, which is characteristic of ferromagnetic order. Thermodynamic parameters, including entropy and Debye temperature, obtained using the quasi-harmonic Debye model reveal signs of lattice stiffening, phonon softening, and anharmonicity. Overall, these findings confirm that AIn_2_S_4_ (A = Fe, Ni) spinels possess thermal stability and strong vibrational features, thereby making them suitable candidates for spintronics and magneto-electronic devices.

## Abstract 2

Mechanical, thermodynamic, and electronic properties for AIn_2_S_4_ (A = Fe, Ni) spinels are examined. Spin-polarized band structures represent half-metallic nature and computed total magnetic moment of 4*µ*_B_ f.u.^−1^ for FeIn_2_S_4_ and 2*µ*_B_ f.u.^−1^ for NiIn_2_S_4_.

## Graphical abstract

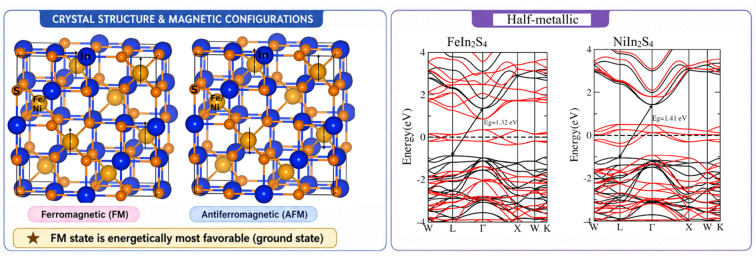

## 1. Introduction

The increasing requirement for high speed, low power consumption, and very miniature electronic devices has pushed traditional charging mechanisms to the limit of their potential, which has resulted in an encouragement towards exploring other methods for storage and computation.^1^ Spintronics has gained prominence as one of the most important methodologies that take advantage of the spin properties of electrons along with their charges for creating devices that are both functional and efficient.^2^ Using spin degrees of freedom, spintronics not only offers non-volatile functionality and high data transfer rates but is also easy to integrate with existing CMOS technologies and has overcome many inherent drawbacks of conventional semiconductors.^3^ Developments such as spin transfer torque, magnetic tunneling junctions, and spin orbit torque switching techniques have, without a doubt, revolutionized logic memory systems and MRAM devices.^4^ Moreover, incorporating novel materials with spintronics like 2D materials, multiferroics,^5^ and topological insulators, among others, has led to some new quantum phenomena and even made spintronics capable of quantum information processing and neuromorphic computing.^6^

Given the importance of speed, sustainability, and multifunctionality in the design of next-generation electronics, it is apparent that spintronics will play a key role in shaping the designs of devices and systems.^7^ It follows that an intricate understanding and precise manipulation of spin-dependent phenomena on the nanometer scale are necessary to develop energy-efficient, scalable, and smart electronic systems for use in energy technology and future computing. The unique electronic structure of half-metallic materials, characterized by insulating or semiconducting behavior for one spin state and metallic behavior for the other, makes them ideal for spintronics applications. As a result, there is perfect spin polarization in half-metallic materials at the Fermi level (*E*_F_), which is a crucial requirement for effective spin sensing and injection in spintronics.^6,8^ Some notable ferromagnets that possess half-metallic properties include manganites, Heusler alloys, spinel chalcogenides, and transition metal oxides.^9,10^

With the successful use of half-metallic alloys as spintronic materials for spin polarization studies, there have been new materials that are being developed to be used in the future spintronics. Such materials, mostly based on AB_2_X_4_ (X = O, S, Se) compositions, possess variable electronic structure band gap and ferrimagnetic characteristics which favor the attainment of half-metallicity.^11^ The spinel ferrites have low scattering and electrical conductivity making them suitable for low loss sensing as well as spin injection.^12,13^ Another feature which makes these spinel chalcogenides an excellent choice for pressure tunable spintronics is their high pressure sensitivity due to soft lattice structures coupled with covalent bonding.^14,15^ Computed mechanical and electronic properties of VCo_2_O_4_ employing first-principles framework used by Ak *et al.*,^16^ confirming half-metallic ferromagnetic character with 10.00*µ*_B_ f.u.^−1^ magnetic moment, having lattice constant value 8.34 Å, and band gaps ranging 1.262–1.286 eV. These compounds also exhibited ductile nature, mechanical stability, and 565 K Debye temperature.^17,18^ In a related study, Özdemir *et al.* examined VRu_2_Br_4_ using comparable computational methodology and found stabilized ferromagnetic phase having magnetic moment value of 10.00*µ*_B_ f.u.^−1^, lattice parameter value of 10.93 Å, and pressure-induced band gaps ranging 0.210–1.718 eV. Particularly, this compound was ductile in nature at ambient pressure *i.e. B*/*G* = 2.48 and *ν* = 0.322 that transitioned as brittle under pressure of 10 GPa, along with 257.22 K Debye temperature.^19,20^

In another study authors examined MnSc_2_Y_4_ (Y = Se, S) and computed static dielectric constants of 6.5 and 8.5, direct band gaps, and large Seebeck coefficients ranging 242–251 µV K^−1^. The systems possess the characteristic features of ferromagnetism due to strong local moments and are mechanically and thermodynamically stable, thus making them viable candidates for use in optoelectronic devices and thermoelectrics.^21–24^ Another study by a different research team on the AIn_2_X_4_ (A = Zn, Cd; X = S, Te, Se) family revealed indirect band gaps ranging from 0.219 eV for ZnIn_2_Te_4_ to 2.294 eV for CdIn_2_S_4_, along with the presence of a strong structure and ultraviolet-visible absorption. Moreover, the figure-of-merit value in the range of 0.725–0.803 at 300 K confirms the suitability of these systems for use in thermoelectrics.^25^ This study gives an overview of the electronic structure, mechanical properties, structural stability, magnetic ordering, and thermodynamics of AIn_2_S_4_ (A = Fe, Ni) spinel structures at various pressures through first principles calculations. It is focused on the aspects of elastic anisotropy, structural stability and dependence of the electronic band gap on the applied pressure, thermodynamic behavior, and magnetism within the framework of quasi-harmonic Debye approximation. This study proves the usability of these spinel structures in magneto-electronic and spintronic device technologies while discussing their multifunctional properties tunable with pressure changes, which have not been widely studied before.

## 2. Computational technique

Density Functional Theory (DFT) calculations were conducted to explore the behavior of spinel chalcogenides AIn_2_S_4_ (A = Fe, Ni) regarding their electronic, structural, magnetic, and thermodynamic properties. Calculations were conducted using the FP-LAPW + lo scheme implemented in WIEN2k software,^26,27^ allowing for very precise predictions concerning transition metal spinels, where the all-electron character of calculations together with flexible modeling of interstitial potentials are important. Ground-state structural parameters were predicted based on the GGA within Perdew–Burke–Ernzerhof for solids (PBEsol) functional, which allows one to obtain improved values of the equilibrium lattice parameters, thanks to its optimized performance for dense materials. In order to examine the electronic structure, the Tran–Blaha modified Becke–Johnson (TB–mBJ) potential was utilized due to known tendency of the standard GGA functionals to underestimate the material's band gap.^28^ The functional choice follows commonly accepted recommendations, where PBEsol functional was used in structural relaxations and mechanical property studies due to its enhanced ability to predict values concerning dense matter, while TB–mBJ was utilized in electronic structure investigations because of its significantly better predictions compared to the traditional GGA or LDA schemes.

Accuracy of the calculated results was achieved through strict optimization of all numerical parameters used. Multiplication of RMT by *K*_max_ was equal to 8 in agreement with convergence requirements of systems with transition and heavy atoms. Muffin-tin radius was equal to 2.36, 2.25, 2.50, and 1.93 a.u. for Fe, Ni, In, and S, correspondingly, to avoid overlapping spheres and have enough of basis function variety in atomic spheres. Energy cut-off for semi-core states was equal to −6 Ry to guarantee their numerical stability. Maximum value of the quantum number determining angular momentums in the partial-wave expansion in muffin-tins, *l*_max_, was taken as 10 to properly account for d- and f-orbitals of the transition metals. Charge density cut-off, *G*_max_, was taken as 18 Ry to obtain proper balance between numerical accuracy and computational efficiency. Brillouin zone sampling was done using a 1000 *k*-point Monkhorst–Pack grid which was enough to achieve the full convergence of total energy; increasing number of *k*-points did not lead to total energy changes. Thermodynamic quantities for AIn_2_S_4_ (A = Fe, Ni) were determined using quasi-harmonic Debye model which is implemented in GIBBS2 code coupled to WIEN2k program.^29^ Hence the approach permits calculation of important thermodynamic properties as functions of pressure and temperature.

## 3. Results and discussion

### 3.1. Structural stability and ground state properties

**Fig. 1:**
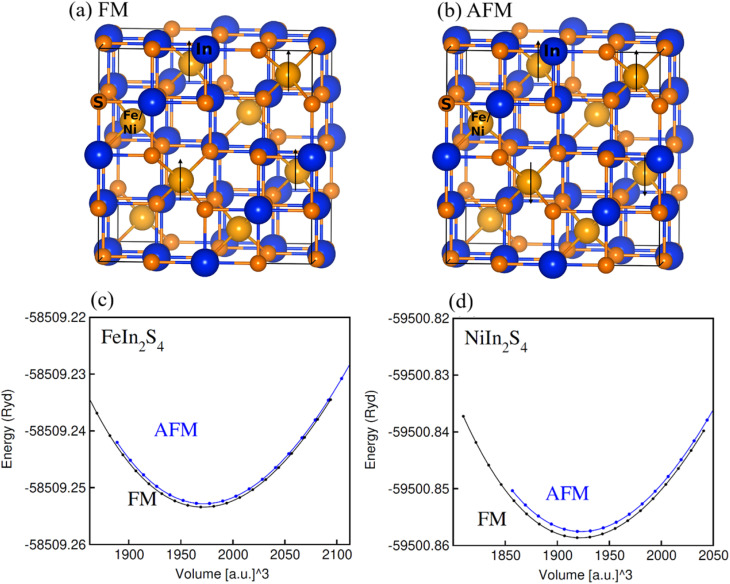
Side view unit cell of spinels AIn_2_S_4_ (A = Fe, Ni) in (a) ferromagnetic (FM), (b) antiferromagnetic configuration (AFM) and volume optimization plots (c) FeIn_2_S_4_, (d) NiIn_2_S_4_ in FM and AFM spin-orientations.

Fig. 1(a and b) shows cubic spinel structure for AIn_2_S_4_ (A = Fe, Ni) in ball-and-stick representations, respectively in ferromagnetic (FM) and anti-ferromagnetic (AFM) configurations. In this framework, Fe, Ni atoms (golden) occupy tetrahedral sites (A), while In atoms (pink) reside at octahedral sites (B), each coordinated by S anions (blue) which makes face-centered cubic unit cell. Both spinel adopt space group of *Fd*3̄*m* along with space group number 227.^30^ Total energy and magnetic ground state calculations were carried out for both antiferromagnetic (AFM) and ferromagnetic (FM) configurations within spin-polarized DFT framework. The energy–volume (*E*–*V*) relationships were computed and are shown in Fig. 1(c) and (d) for FeIn_2_S_4_ and NiIn_2_S_4_, respectively. Ferromagnetic configuration steadily yields lower total energy compared to the antiferromagnetic state, indicating a clear thermodynamic preference for ferromagnetic ordering. Calculated values of energy difference is reported in Table 1. The magnitude of this energy difference establishes the relative stability of FM phase.

**Table 1:** The ground state lattice constants *a*_0_ (Å) and bond length at 0 GPa, 2 GPa and 4 GPa, bulk modulus *B*_0_ (GPa), enthalpy of formation Δ*H*_f_ (eV), and elastic parameters, calculated for AIn_2_S_4_ (A = Fe, Ni)

Compounds	Pressure (GPa)	*a*_0_ (Å)	*B*_0_ (GPa)	Δ*E* = *E*_AFM_–*E*_FM_ (meV)	Δ*H*_f_ (eV)	Bond length (Å)
FeIn_2_S_4_	0	10.53	155.79	48.22	−0.91	Fe–S 2.838
		Exp. = 10.62^a^				In–S 2.933
	2	10.47	—	—	—	Fe–S 2.822
						In–S 2.916
	4	10.40	—	—	—	Fe–S 2.803
						In–S 2.897
NiIn_2_S_4_	0	10.45	155.79	39.82	−0.80	Ni–S 2.933
		Exp. = 10.51^a^				In–S 2.586
	2	10.39	—	—	—	Ni–S 2.916
						In–S 2.571
	4	10.32	—	—	—	Ni–S 2.897
						In–S 2.554

a Ref. 36.

Formation enthalpy (Δ*H*_f_) was subsequently computed to further assess thermodynamic stability, using the relation shown below.^31^

1 Δ*H*_f_ = *E*_Total_(A_*l*_In_*m*_S_*n*_) − *lE*_A_ − *mE*_In_ − *nE*_S_

In this expression, *E*_Total_ denotes the total ground state energy of the spinel compound, while *E*_Fe_, *E*_Ni_, *E*_In_, and *E*_S_ represent the total energies of the isolated elemental species in their most stable configurations. Coefficients *l*, m, and *n* indicate the number of (Fe, Ni), In, and S atoms, respectively.

In Table 1, calculated formation energies are listed, which are negative for both spinels, confirming thermodynamic stability relative to the elemental constituents. The less negative value for NiIn_2_S_4_ compared to FeIn_2_S_4_ reflects a modest decrease in stability upon chalcogen substitution, consistent with weaker In–S bonding arising with greater ionic radius of Fe^2−^ relative to Ni^2−^ and the resulting increase in bond length (see Table 1). Equilibrium parameters for the structure along with bulk modulus *B*_0_ and lattice constant *a*_0_, were determined by volume optimization within the FM state utilizing Murnaghan equation of state in conjunction with *E*–*V* data obtained with PBEsol functional.

The calculated *a*_0_ show that there is a systematic decrease in lattice constant of FeIn_2_S_4_ and NiIn_2_S_4_ with an increase in pressure between 0 and 4 GPa, which is the compression of the lattice. Even though the reported nearest-neighbor bond lengths (Fe–S = 2.8379 Å, (In–S = 2.9329 Å) in FeIn_2_S_4_) do not change significantly when listed in the Table 1, the overall volume decrease would suggest increased orbital overlap between cation d-states (Fe/Ni) and anion p-states (S). This further hybridization, which makes the bandwidth of each band of the conduction and valence band broader. As a result, the splitting of bonding and antibonding changes with pressure, which tends to lead to increase in the band gaps. Additionally, changes in the d-orbital splitting due to pressure-induced changes in the crystal field environment around transition metal ions (Fe^2+^/Ni^2+^), also can alter the d-orbital splitting, which also contributes to the shifting of electronic states near the Fermi level. Therefore, the combination of an increase of the orbital overlap, a change in the hybridization and a change in the crystal field all serve to provide a consistent account of the observed behavior of the pressure-dependent electronic behavior in these spinel compounds. Moreover, reduction in these distances enhances orbital overlap and shifts the bonding/anti-bonding states in light of work reported on Co_2_−*x*V_*x*_FeGe full-Heusler alloys, LiScNiGe for optoelectronic applications and TaAlCuCo investigated *via* DFT.^32–34^ This now provides a strong physical basis for the observed band gap increased.

As anticipated, the lattice constant increases from FeIn_2_S_4_ in comparison with NiIn_2_S_4_ due to the larger ionic size of indium, which expands the average interatomic spacing. This is similar to what has been observed in related systems like MnSc_2_S_4_ and MnSc_2_Se_4_.^35^ Likewise, the volume modulus is reduced; that is, the compound is more compressible than indium. This correlation between bulk modulus and lattice parameter is in accord with the general knowledge of solids: increased expansive unit cell correlates with a decrease in mechanical strength – *i.e.*, a decrease in the strength of the interactions between atoms in the solid. The calculated lattice constant for FeIn_2_S_4_ and NiIn_2_S_4_ is consistent with earlier reported experimental results.^36^

**Fig. 2:**
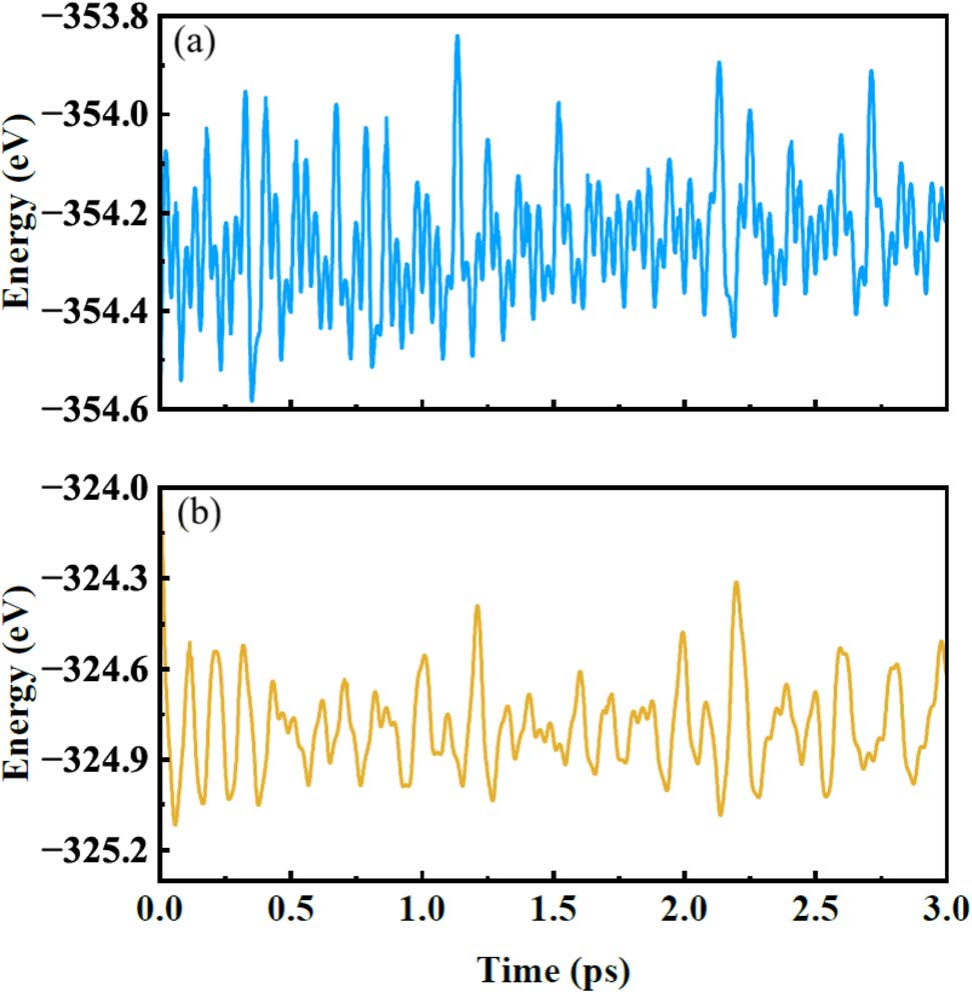
AIMD plot for spinel structures of (a) FeIn_2_S_4_ and (b) NiIn_2_S_4_.

Thermal stability is one of the important properties which should be analyzed before applying any material. Here, *ab initio* molecular dynamics (AIMD) calculations were carried out to study the thermal behavior of hydrides. The AIMD calculation was carried out in the canonical NVT ensemble using the Nosé thermostat method to ensure accurate energy control during the simulation time period.^37^ AIMD simulations were performed at 300 K for the assessment of structural stability at finite temperatures. Fig. 2(a and b) shows the energy variation trend from 0 to 3 ps simulation time for total free energy. These energy values oscillate slightly about their equilibrium value without undergoing any structural distortions. This ensures the stable nature of the systems studied. FeIn_2_S_4_ and NiIn_2_S_4_ have shown oscillation in their energy values at 300 K and it ensures structural stability. Therefore, the spinel structures of FeIn_2_S_4_ and NiIn_2_S_4_ can be expected to have improved stability at low temperatures.

### 3.2. Mechanical properties and elastic anisotropy

Elastic response for FeIn_2_S_4_ and NiIn_2_S_4_ was characterized by determining the independent second-order elastic constants *i.e. C*_11_, *C*_12_, and *C*_44_, which are crucial for describing mechanical behavior of crystals (cubic) and determining the principal elastic moduli.^38^ Computed elastic constants fulfil stability criteria proposed by Born for cubic crystals *i.e. C*_11_ > 0, *C*_11_ − *C*_12_ > 0, *C*_11_ + 2*C*_12_ > 0, confirming mechanical stability under ambient conditions. Bulk modulus (B), Young's modulus (*E*) and shear modulus (*G*) were derived using Voigt–Reuss–Hill averaging of elastic constants and reported in Table 1 at 0 GPa. Obtained Bulk moduli agree well with values from geometry optimization, validating mechanical robustness for both compounds and indicating strong resistance to the volumetric compression. Elastic behavior of studied spinels also have an apparent pressure-sensitive development, which illustrates the increased mechanical stability and hardness during compression. In the case of FeIn_2_S_4_, the elastic constants increase with pressure, and thus the material offers better resistance to longitudinal and shear deformations. In line with this, the bulk modulus (*B*) increases with compression in that the bulk modulus (*B*) increases, which is in line with increased incompressibility (see Table 2). In the case of NiIn_2_S_4_, the same tendency is observed but with some more complexities. Although *C*_11_ rises considerably with pressure, *C*_44_ decreases slightly with increased pressure, implying that there is anisotropic resistance to shear deformation.

**Table 2:** Calculated values of elastic constant, bulk modulus (*B*), Shear modulus (*G*), Young's modulus (*Y*), Poisson's ratio (*υ*) and Pugh ratio (*B*/*G*) for spinels FeIn_2_S_4_ and NiIn_2_S_4_ at different pressure 0 GPa, 2 GPa and 4 GPa

	Pressure (GPa)	*C*_11_ (GPa)	*C*_12_ (GPa)	*C*_44_ (GPa)	*B* (GPa)	*G* (GPa)	*Y* (GPa)	*ν*	*B*_0_/*G*
FeIn_2_S_4_	0	142.6	38.8	27.79	73.43	35.78	92.34	0.290	2.052
	2	146.9	46.84	30.44	80.21	37.17	96.61	0.299	2.157
	4	152.5	54.25	37.33	87.01	41.68	107.8	0.293	2.087
NiIn_2_S_4_	0	136.5	55.54	36.68	82.53	38.15	99.16	0.301	2.163
	2	147.7	60.17	29.84	89.36	34.81	92.42	0.328	2.567
	4	182.2	54.67	29.15	97.18	40.12	105.8	0.318	2.422

Ductile or brittle characteristics were assessed by Pugh ratio (*B*/*G*) and Poisson's ratio (*υ*). Materials with *B*/*G* > 1.76 and *ν* > 0.27 exhibit ductility. Computed values at 0 GPa are *B*/*G* = 2.05 and *υ* = 0.29 for FeIn_2_S_4_, and *B*/*G* = 2.16 and *υ* = 0.30 for NiIn_2_S_4_, both well above the critical thresholds and indicating ductile response.^39^ Generally, both compounds are stiffer under mechanical stress and retain ductile characteristics under pressure, with NiIn_2_S_4_ having a higher sensitivity to pressure and being anisotropic in behavior. This behavior is favorable for applications demanding mechanical resilience and fracture resistance under operational stress, such as thermo-mechanical devices and robust electronic platforms.^40,41^ The mechanical characteristics of AIn_2_S_4_ (A = Fe, Ni) are consistent with established trends in ductile systems, including recently characterized MAX phases and solid solutions, affirming their suitability for device applications and spintronics.

**Fig. 3:**
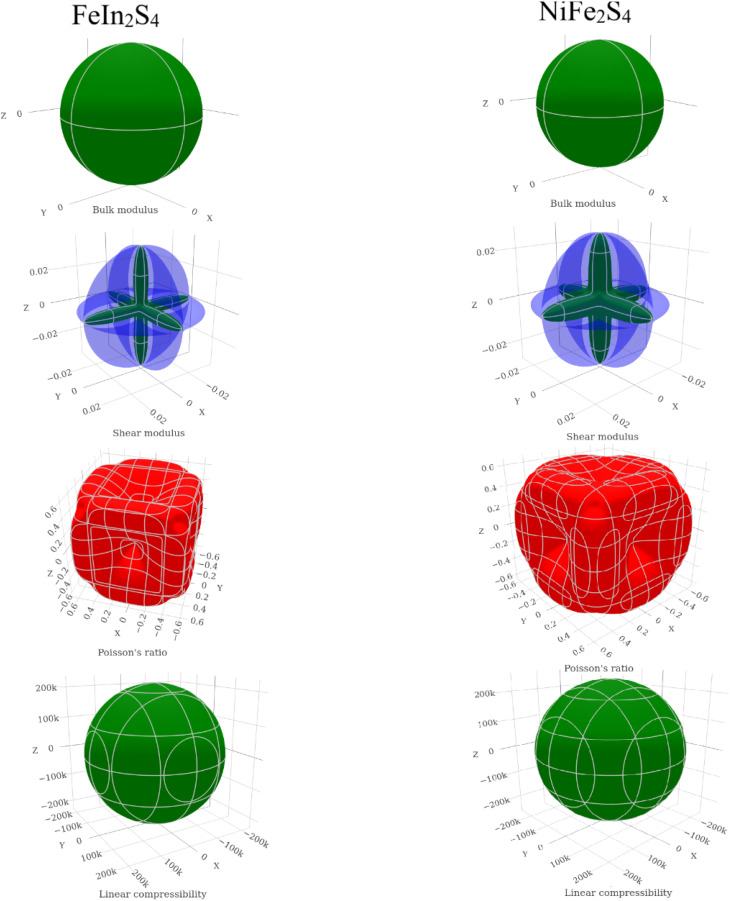
3-D representation of bulk modulus (*B*), shear modulus (*G*), Poisson's ratio (*v*), Pugh's ratio, and Hardness for AIn_2_S_4_ (A = Fe, Ni) at 0 GPa.

Elastic anisotropy is an intrinsic property of crystalline materials and can alter all their properties, such as deformation, mechanical stability, and movement and transfer of phonons through the materials.^42^ This anisotropy is manifested in the variation of elastic properties (elastic moduli) with direction in the crystal lattice. This is not the case with isotropic materials, which behave mechanically the same regardless of direction. To check for a better understanding of the isotropic nature in AIn_2_S_4_ compounds, where A is Fe or Ni, one made three-dimensional plots of Young's modulus (*Y*), linear compressibility (*β*), Poisson's ratio (*υ*), and shear modulus (*G*) at zero pressure. The surfaces are depicted in Fig. 3. If it were an ideal isotropic solid, these shapes should look something like spheres. All parts of a sphere are distorted and directly point to the presence of anisotropy. FeIn_2_S_4_ does not cling to spherical shapes, particularly with *G* and *β*, and it should be fairly obvious that this compound is highly anisotropic. Looking at the ratios in isotropic materials, they are all identical to one. However, the values of FeIn_2_S_4_ are far from unity, indicating the strong level of anisotropy in both Young modulus and shear response. The NiIn_2_S_4_ maintains the close ratio of 1 : 2. Its surface has only slight deviations from a sphere, indicating that NiIn_2_S_4_ is far more homogeneous in its elastic properties.

The Fe–S and In–S bonds are the ones that are exerting different pulls in different directions, thus introducing stronger elastic anisotropy in FeIn_2_S_4_. This causes significant variation in the resistance to push or pull, varying from place to place within the crystal.^43^ But the situation is different if we replace Fe with Ni, such as NiIn_2_Se_4_. Iron (Fe) atom are larger than Ni atom, and they also have higher polarizability, which softens the lattice and increases its flexibility. This softness makes the elastic response uniform in the entire crystal and reduces the anisotropy. The choice of chalcogen reflects not only the geometry and size of the crystal structure but also the directionality of the mechanical properties. Directional mechanical effects will be of interest to those who are interested in the characteristics of these compounds under stress or in devices. This is important for applications such as magnetostriction, spintronics, or pressure-tuned optoelectronics, where the directionality of the response to a change in magnetic field or pressure actually determines the efficacy of the material.

### 3.3. Pressure-modulated electronic properties

**Fig. 4:**
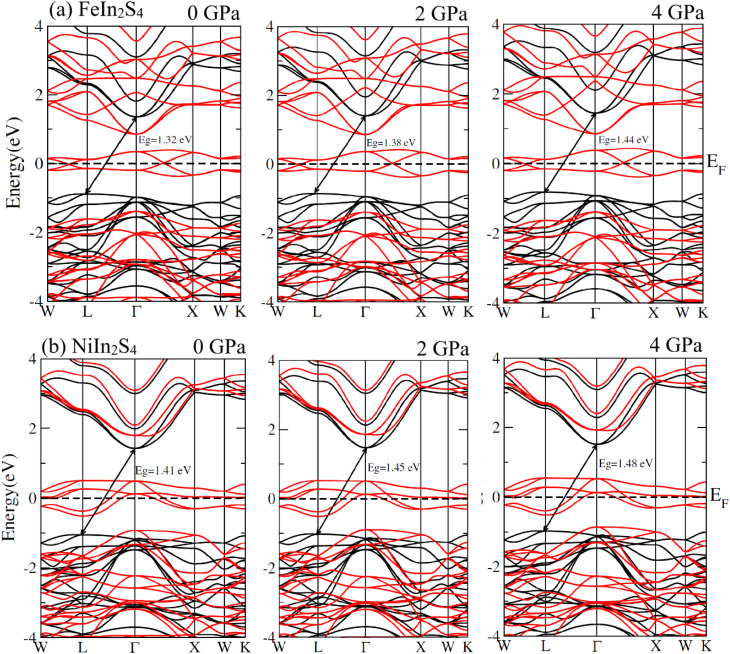
The electronic band structures for spinels (a) FeIn_2_S_4_ and (b) NiIn_2_S_4_ in spin up (black line) and spin down (red line) configurations at pressure 0 GPa, 2 GPa and 4 GPa computed with mBJ potential.

**Fig. 5:**
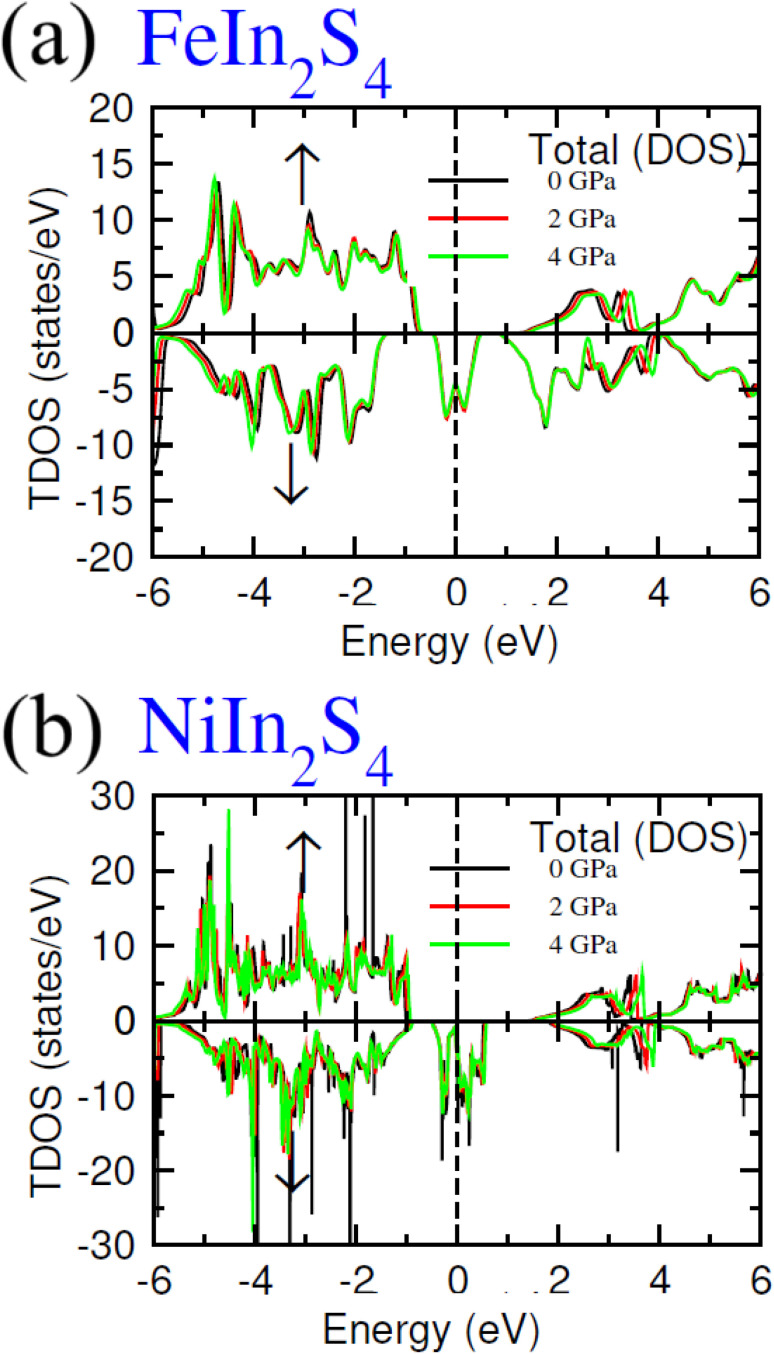
Total DOS for spinels (a) FeIn_2_S_4_ and (b) NiIn_2_S_4_ at different pressures in spin-up (↑) and spin-down (↓), computed with mBJ potential.

**Fig. 6:**
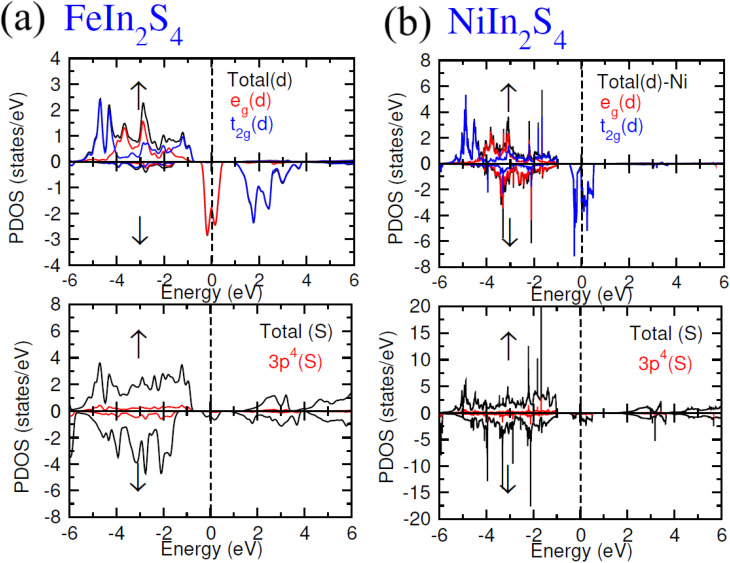
Partial DOS for spinels (a) FeIn_2_S_4_ and (b) NiIn_2_S_4_ at 0 GPa in spin-up (↑) and spin-down (↓), computed with mBJ potential.

The properties of a material are determined by its electronic structure, and its suitability for a particular device depends on its electronic structure.^44,45^ The band gap is a particularly important parameter, directly dictating performance among all parameters, especially in the field of memory, advanced spintronics, and electronics. Such effects are not only on the efficiencies of the device, but also on the application scope of the device, such as signal processing, data storage, and spin control when the band gap controls charge and spin transport. We calculated the electronic properties of materials AIn_2_S_4_ based on TB-mBJ potential as displayed in Fig. 5 and 6, with A = Fe and Ni, respectively. It is found that FeIn_2_S_4_ has an indirect band gap at the *L*–*Γ* point when the pressure is zero (see Fig. 4). It behaves like a semiconductor in the up-spin configuration and more like a metal in the down-spin configuration. Carriers are more or less mobile as move along the band structure at the high-symmetry points in the Brillouin zone, and there is ample band dispersion—most notably within the conduction band, indicating just the right amount of spread or localization. The spin-up (black) and spin-down (red) states are clearly different in the immediate vicinity of the Fermi level. This split is a good indicator of the spin polarization, and that magnetic ordering is found in this structure. This type of behavior is due to exchange interactions involving Fe 3d electrons, typical of magnetic spinels. The band structure for this material at atmospheric pressure points towards a localised nature for the carriers.

At 2 GPa (Fig. 4a), can be seen that the material begins to exhibit slight changes in response to pressure. This results in widening of the indirect band gap due to the shift upwards of the conduction band minimum (CBM) in the up-spin channel and the small shift upwards of the valence band maximum (VBM) in the down-spin channel.^46^ The widening of the band gap indicates increased hybridization of the orbitals, increased delocalization of the electrons, both of which resulted from the compression of the lattice. As the pressure increases, the Fe S–In coupling strengthens, the Fe–S–In orbitals at the edges of the conduction band being particularly prominent.

At 4 GPa, the bandstructure has more changes, with the indirect band gap (EIG) continuing to increase. The CBE is pushed further up while the VBM shifts up negligibly. Well, that makes the gap remain indirect. Even under this high pressure, magnetic exchange interactions are still intact and stable because the spin polarization is found everywhere in the Brillouin zone. That suggests that the primary magnetic coupling (Fe–S–Fe super-exchange in the spinel lattice) does not vanish under the squeeze of the crystal.

This is a type of electronically-active pressure-induced response that allows us to fine-tune the dynamics of the spins and charges: FeIn_2_S_4_ is a worthwhile candidate for magneto-functional and spintronic devices operating under pressure. Fig. 4b shows the spin-polarized band structures for NiIn_2_S_4_ at 0, 2, and 4 GPa, using the TB–mBJ potential. Plot of energy states with the main high symmetry points of the FCC, B-zone map. Can observe how the spin-polarized bands evolve, capturing the electronic/magnetic response of the compound when subjected to pressure. When *P* = 0, NiIn_2_S_4_ is an indirect semiconductor (*L* is the point in the valence band maximum position, and *Γ* is the position of the minimum of the conduction band).

The majority of the band gap widens with increasing pressure applied up to 2 GPa. As interatomic distances approach the FeS overlap also rises, and, as mentioned, the In-3d, and the S-4p orbitals are closer together and exhibit a stronger hybridization. The up-spin channel has a conduction band minimum shift up, and the down-spin channel is also shifted up. It creates this kind of response that enhances the thermodynamic stability and increases the electronic conductivity.^47^ Impress pressure exceeding 4 GPa, the band gap widens up again. The monotonous increase of the indirect band gap at all pressure levels as well as the presence of half-metallic properties and spin asymmetry, make NiIn_2_S_4_ a pressure-tunable quantum material in particular. Excellent compressibility; both the electronic and magnetic properties can be adjusted using fine controls.

The spin polarization (SP) is the necessary condition for half-metallic ferromagnetism and guarantees the complete polarized degree of spin. In the spinels studied, the spin (↑) channel is metallic, and states cross the *E*_F_ level, denoted as *D* (↑), whereas in the spin (↓) channel, the *E*_F_ level lies within the forbidden zone, with *D* (↓) = 0. The numerical values of *D* (↑), *D* (↓), and SP are presented in the Table 3. Therefore, according to the formula given below, the studied materials are completely spin-polarized.2
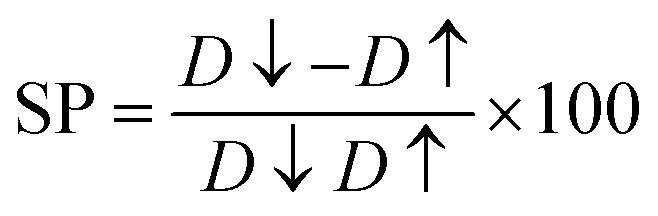


**Table 3:** Quantitative spin-polarization at the Fermi level for spinels FeIn_2_S_4_ and NiIn_2_S_4_

Compound	0 GPa	2 GPa	4 GPa
FeIn_2_S_4_	—	—	—
*D* (↑)	0.42	0.47	0.51
*D* (↓)	0.0	0.0	0.0
SP	1.0	1.0	1.0
NiIn_2_S_4_	—	—	—
*D* (↑)	0.47	0.51	0.60
*D* (↓)	0.0	0.0	0.0
SP	1.0	1.0	1.0

In Fig. 5 and 6, the density of states (DOS) analysis is further progressed on FeIn_2_S_4_ and NiIn_2_S_4_, respectively, for the purpose of a pressure study. Fig. 5(a and b) displays the total DOS of both compounds at 0, 2, and 4 GPa in the spin-up and spin-down channels, respectively. Take FeIn_2_S_4_: At zero pressure, there is a clear separation between the spin up and spin down states – there is strong spin polarization and large exchange splitting around the Fermi level. At the Fermi energy, the spin-down channel stays inside the band gap, and the spin-up channel remains semiconducting. That implies that real-spin-dependent behaviour is observe for electrons. As the pressure increases to 2–4 GPa, the corresponding band edges of both spin states move, and the band gap gradually widens.^48^ This increase in the band gap, accompanied by increased density of states within the band gap at the Fermi level, suggests an increased degree of electronic delocalisation and strengthened pressure-induced magnetic exchange interactions, particularly *via* Fe–S–Fe super-exchange pathways, arising from the lattice squeezing.

As seen in Fig. 5(b), NiIn_2_S_4_ exhibits less spin polarization and lower exchange splitting as compared to the other cases. On both channels of spins, the total density of states at the Fermi level is not zero at zero GPa. This is in line with its weak metallic or semi-metallic properties. Upon application of pressure, no significant change in the density of states is observed; that is, NiIn_2_S_4_ maintains its low-spin status. This is because there is an increase in the width of the band for the new materials, and reduced mixing of Ni-3d and S-4p states. Here, the lower value of the electronegativity of indium and the larger atomic radius take a role and are responsible for blocking magnetic-exchange pathways.

Next, observe Fig. 6(a and b). The partial density of states (PDOS) at zero GPa decomposes into the contributions from the individual orbitals of Fe/Ni atoms and the chalcogen elements, thus showing an exact breakdown of how the components contribute to the electronic density. The analysis of Fe 3d states projected into the orbitals of the chalcogen p orbital provides a pictorial representation of the mixing and the hybridization of these states. Chemical bonding, charge distribution, and the influence of each atom on the electronic and magnetic properties are clearly seen.

In FeIn_2_S_4_, the Fe 3d states split into the t_2g_ and e_g_ states—the latter of which will tend to dominate near the Fermi level. Exchange splitting is noteworthy: the eg orbitals of up-spins are located just below the t_2g_ orbitals, while the Fermi level is above *E*_F_, aligning with an up-spin Ni^2+^ configuration. The 3p-orbital of sulfur clearly interacts with the Fe-3d valence region (from about −5 eV to 0 eV), in this region, there is quite a bit of p–d covalence. But this is significant since this directly influences the electronic transport and magnetic ordering.

The Ni-3d orbitals are spread out more than normal in NiIn_2_S_4_. Less exchange splitting, more diffuse-like the bands look. That would suggest that the spin interaction is weaker and the electronic states have more overlap in the conduction band. The energy range of the S-3p orbitals overlaps quite extensively as well, but they do not “mix up” with the Ni-3d states, so the wavefunctions remain less mixed up and more spread. Because of this, the magnetic moment doesn't stick closely to the Ni atoms. The global polarization of the material decreases, and this brings it closer to a weakly magnetic or even semi-metallic state.^49^ The wider band gap for the density of states, and the lower degree of separation of the orbitals both indicate that the ligand field is less strong, which implies that magnetic ordering is less likely in the nickel sulphide than iron sulphide.

This electronic structure analysis reveals that the FeIn_2_S_4_ has a stronger spin polarization feature and half-metallic property under pressure, while the NiIn_2_S_4_ has a more delocalized, weakly magnetic property. Significant changes occur in the electronic structure of the two spinel structures under applied pressure, but far larger in the sulfide structure. From the evaluation of lattice compression, it is found that the magnitude of interatomic distance is decreased, and the magnitude of orbital overlapping between Fe 3d state, In 3d state, and S 3p state is increased in Fe-based sulfides. This increased overlap means a greater electronic interaction and better charge delocalisation in the lattice. This results in the enhancement of magnetic exchange interactions in the material by a pressure-driven hybridization, thereby stabilizing the spin-alignment and enhancing its overall magnetic character. Such conflicting responses highlight an essential part of the chalcogen anion to controlling competition between electron delocalization and localization. The results are significant for developing spinel having engineered electronic and magnetic properties for spintronics, magneto-electric, and energy conversion applications.

### 3.4. Pressure induced magnetic characteristics

Magnetic properties play a central role in how spinel compounds work in spintronics and magneto-electronic devices. To explore this, we calculated both the total and site-resolved magnetic moments for AIn_2_S_4_ (where A is Fe or Ni) using density functional theory. Results can be found in Table 4, reported in Bohr magnetons (*µ*_B_).

**Table 4:** The spin up bandgap (*E*_g_ (eV), total, and the local magnetic moments (in Bohr magneton) calculated for AIn_2_X_4_ (A = Fe, Ni)

	Pressure (Gpa)	↑*E*_g_ (eV)	*M*_Total_ (*µ*_B_)	*M*_X_ (*µ*_B_)	*M*_In_ (*µ*_B_)	*M*_S_ (*µ*_B_)
FeIn_2_S_4_	0	1.32	4.00058	3.4141	0.0159	0.0662
	2	1.38	4.00062	3.391	0.0176	0.0678
	4	1.44	4.00060	3.368	0.0193	0.0690
NiIn_2_S_4_	0	1.41	2.00030	1.4719	0.0023	0.0881
	2	1.45	2.00032	1.4577	0.0011	0.0890
	4	1.48	1.99941	1.4209	0.0004	0.0931

In spinel FeIn_2_S_4_, the band gap keeps rising as pressure increases—starting at 1.32 eV with no pressure, and reaching 1.44 eV under 4 GPa. This shift comes from higher crystal field splitting and changes in p–d hybridization as the structure compresses.^50^ The material's total magnetic moment stays steady at about 4*µ*_B_, so its ferromagnetic ground state remains quite solid. There's a small decrease in the Fe-site magnetic moment, from 3.4141*µ*_B_ down to 3.368*µ*_B_, while the magnetic moment on the In and S sites ticks up a bit. That suggests spin density shifting around, probably because of more orbital overlap. The causes of the drop is probably the covalent interactions and hybridization between the chalcogen p and the Fe or Ni d orbitals which is usually called hybridization-induced magnetic polarization.^51^

With NiIn_2_S_4_, show similar pressure effect, but it's subtler. The band gap rises from 1.41 to 1.48 eV as pressure grows, but the change is smaller compared to FeIn_2_S_4_. The total magnetic moment hovers near 2*µ*_B_, showing its magnetic ordering holds steady. In both cases, the induced moments arise from super-exchange along Fe–S–Fe pathways, letting some spin density spread onto neighboring sites—especially the anions.^52^ The interstitial regions have additional magnetic moments, of about 0.579*µ*_B_ in FeIn_2_S_4_ and 0.361*µ*_B_ in NiIn_2_S_4_ for zero pressure, which indicate strong spin delocalization. This is because Fe-3d states are more extended in space, and in-4p orbitals are more extended in space than the small S-3p orbital. The increased crystal size allows for greater overlap of neighbouring wave functions, which results in increased orbital hybridization in the crystal. As these interactions become more powerful, the electrons become more delocalized, and the magnetic exchange between Fe and Ni in sites becomes even closer.

### 3.5. Thermodynamic analysis

Using the quasi-harmonic Debye model and the GIBBS2 code, the thermodynamic properties of the AIn_2_S_4_ (A = Fe or Ni) system were considered as functions of pressure and temperature. By using this method, we can determine other important thermal properties such as Debye temperature (*θ*_D_), entropy (*S*), thermal expansion coefficient (*α*), and heat capacity (*C*_V_). Temperatures covered were from 0 to 1000 K, and pressures between 0 and 4 GPa.

The results of the thermodynamics obtained up to 600 K are from calculations carried out under the quasi-harmonic Debye approximation. The selected temperature range was found to span the overall thermal response and trends observed in lattice dynamics, including such as entropy, lattice heat capacities, and lattice Debye temperature.^53^ The physical limitations involved in the melting and Curie temperatures of the compound are, however, very much constraining the quantitative accuracy of those results. Immediately after these limits, anharmonic effects and magnetic transitions become increasingly pronounced, making the quasi-harmonic approximation a less useful tool for the accurate modeling and prediction of AIn_2_S_4_ (A = Fe, Ni). The values so determined, therefore, can only be considered theoretical values beyond these temperatures, and not final experimental values. The quasi-harmonic approximation is no longer fully applicable in this extreme case of high temperature, where more significant anharmonic lattice vibrations, real conditions, and induced phase transitions and magnetic fluctuations may occur. Thus, the computed trends give a qualitative view of the thermodynamic response of a material and are not necessarily quantitatively correct representations of experimental results. These thermodynamic quantities shed light on mechanical robustness, anharmonic vibrational behavior, and phonon dynamics, which are critical for assessing applicability.^54^

**Fig. 7:**
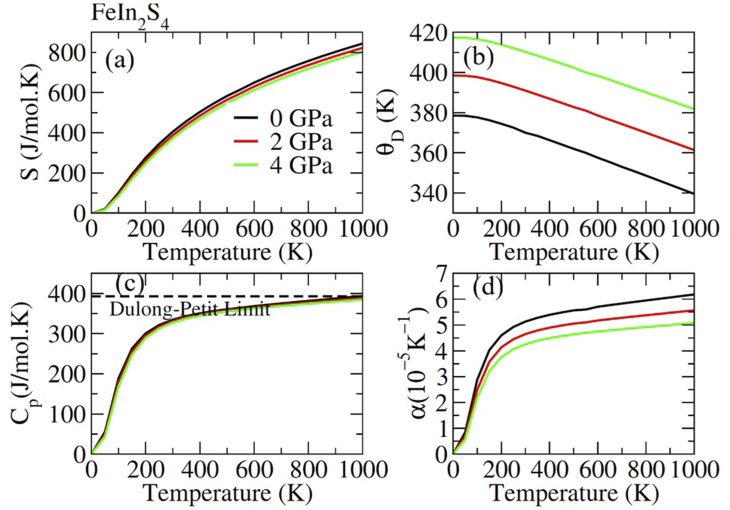
Calculated values of (a) entropy, (b) Debye temperature (c) *C*_p_ and (d) coefficient of thermal expansion (*α*) for spinel FeIn_2_S_4_ at different pressure.

**Fig. 8:**
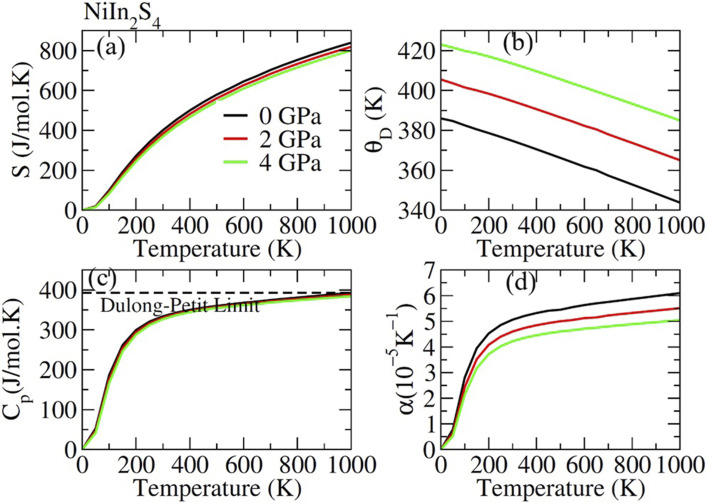
Calculated values of (a) entropy, (b) Debye temperature (c) *C*_p_ and (d) coefficient of thermal expansion (*α*) for spinel NiIn_2_S_4_ at different pressure.

The role of entropy in considering the amount of disorder within a crystal lattice material and filling of the phonon modes forms the central role of the concept. Looking at Fig. 7 and 8(a), one can observe that the entropy increases sharply with the increase of temperature, becoming close to zero at lower temperatures (that is, at absolute zero temperatures). This corresponds to the third law of thermodynamics! the interesting thing is the steepness of this rise at low temperatures, where the phonon modes become excited rapidly. However, as the temperature rises, the curve levels off as any number of the modes are occupied.

Pressure really stands out as a factor in NiIn_2_S_4_, especially when the temperature rises. At 300 K and zero pressure, entropy hits 410 J mol^−1^ K^−1^ for FeIn_2_S_4_ and 398 J mol^−1^ K^−1^ for NiIn_2_S_4_—just check Fig. 7 and 8(a). The trend from Fe to Ni shows a clear boost in the available vibrational degrees of freedom and, thanks to the heavier atomic mass, spinel compounds like NiIn_2_S_4_ react more strongly. These changes are smiliar to the behavior described in earlier studies.^52^

The above relationship between entropy and temperature and pressure is closely related to a material's vibrational properties and resistance of the lattice, as described by the Debye temperature, *θ*_D_. The Debye temperature changes very slightly at low temperatures, as can be seen from Fig. 7 and 8(b). Then, after around 280 K, the FeIn_2_S_4_ drops pretty linearly and NiIn_2_S_4_ drops after 220 K. That is a first indication of phonon softening and increasing anharmonicity at high temperatures.

With the increase in pressure, the Debye temperature of FeIn_2_S_4_ and NiIn_2_S_4_ decreases almost linearly representing the reduction in average phonon frequency and continuing softening of the lattice. Additionally, phonon frequencies suddenly drop rapidly with pressure, totally matching the expansion in entropy. The chemical and mechanical changes that occur together provide evidence that the crystal becomes more ordered and mechanically rigid when it is compressed. For FeIn_2_S_4_ and NiIn_2_S_4_, the Debye temperatures are 378 and 386 K, respectively, when pressure is zero, and the temperature is 300 K. There exists a characteristic inverse relationship between the higher Debye temperature and lighter atomic mass. This is characteristic of the pattern with decrease in the frequencies of phonons generated by lighter atoms and the resulting tighter bond, which cascades throughout the material, thereby altering the material's resulting thermal conductivity and entropy. The increase in phonon frequency with pressure can be understood from the increase in the restoring forces between atoms, while the increase in lattice vibrations and phonon softening with temperature is responsible for the decrease in *θ*_D_. From a quantitative point of view, the difference in *θ*_D_ seen in the present system is comparable to those found in the recently studied perovskite oxides XWO_3_ (X = Sr, Cd),^53^ for which the quasi-harmonic Debye approximation also predicts that the change in *θ*_D_ is monotonic with both temperature and pressure, in the range 0–1000 K and 0–8 GPa. This similarity in behaviour suggests that the thermodynamic response of these compounds is controlled by similar lattice-dynamical effects which are common to oxide perovskites.

The heat capacity *C*_p_ is closely related to both entropy and the Debye temperature. At very low temperatures, *C*_p_ follows the characteristic *T*^3^ dependence predicted by Debye theory for phonons. This is the same *T*^3^ behavior commonly observed in low-temperature lattice heat capacity curves.^54,55^ As the temperature rises, the material comes nearer and nearer to the Dulong–Petit limit: there, all phonon modes are completely activated, filled with thermal energy. The transition from quantum to classical behaviour is striking below a little under 220 kelvin (K). Following this, the rate of increase in *C*_p_ gets even lower but eventually levels out. Pressure has little effect; it shifts the position of *C*_V_ slightly by compressing the lattice.^56^ The *C*_p_ values are found, at zero pressure and 300 K, as 336 J mol^−1^ K^−1^ for FeIn_2_S_4_ and 322 J mol^−1^ K^−1^ for NiIn_2_S_4_. The increase is consistent with the increase in entropy and structure for both materials.

The coefficient of thermal expansion (*α*) was studied for its behavior in volume change with temperature under different pressures. As seen in Fig. 7 and 8(d) it is highly dependent on both temperature and pressure; it is actually following *C*_p_ closely, but pressure's effect on *α* is higher to an extent.^57,58^ The rate of increase in *α* is great at low temperature; this indicates that lattice anharmonic vibrations increase with rising temperature. As the temperature continues to increase, the *α* begins to level off and approaches a near constant value similar to what approaches the Dulong–Petit value.^59^

At 0 GPa and 300 K, *α* is 5.06 × 10^−5^ K^−1^ for FeIn_2_S_4_ and 3.94 × 10^−5^ K^−1^ for NiIn_2_S_4_. The larger the value of *α* for FeIn_2_S_4_, the greater the atomic size of Fe than Ni and its polarizability due to this, there is less rigidity in the lattice so that more of the vibrations become anharmonic, which increases the extent of expansion when heated. When pressure is applied, the values of *α* are both sharply reduced for both compounds. This is analogous to the way condensed matter typically works: push down on the lattice and reduce these anharmonic effects and vibrations, and, in turn, reduce the structure's stiffness and the temperature coefficient of its thermal expansion.

### 3.6. Optical analysis

Understanding the dielectric function is necessary for investigating the interaction of materials with electromagnetic fields. It is important to gain a profound insight into the behavior of the material when interacting with electromagnetic waves since this knowledge is required for the implementation of many optical applications such as photovoltaic cells, optical sensors, and LEDs. Additionally, the dielectric function can be considered a good indicator of the structure and behavior of the material under investigation.

In this paper, the values of the complex dielectric function were estimated based on the use of Kramers–Kronig relations. The formulas for estimating the real and imaginary parts of the dielectric function can be presented as^60,61^3
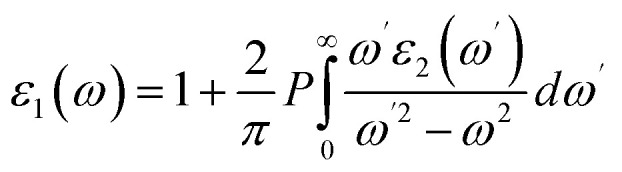
4
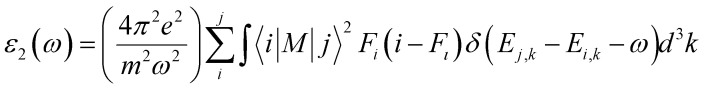
In this case, *ω* is the angular frequency, while *P* stands for the Cauchy principal value of the integral. *M*_*if*_ stands for the transition dipole moment matrix elements in which indices *i* and *f* refer to the initial and final electronic states correspondingly. In addition, *F*_*i*_ is the Fermi–Dirac distribution function and *E*_*i*_ refers to the energy of electrons in the ith state. Finally, the wave vector is represented by *k*. The properties of the material in relation to light can be characterized by its complex-valued dielectric constant. The first component of the latter, *ε*_1_(*ω*), determines how the material responds to the electric field; on the contrary, the second component, *ε*_2_(*ω*), is related to the absorption or attenuation of radiation.

**Fig. 9:**
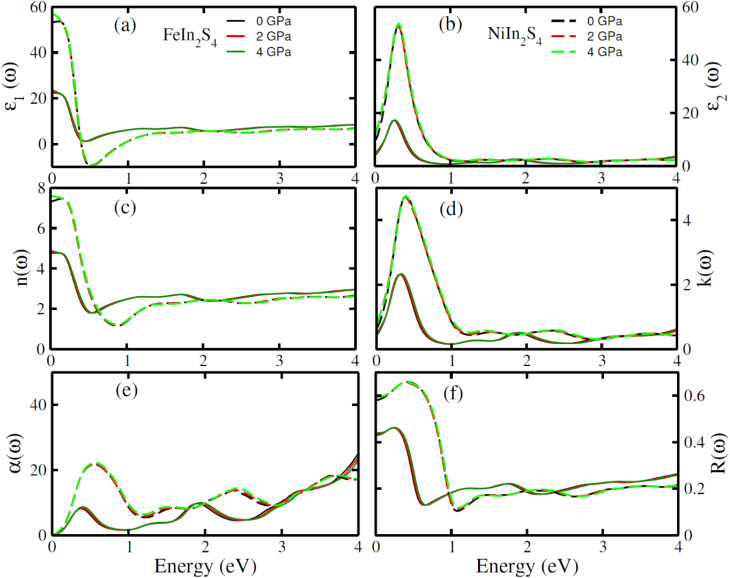
Calculated values of (a) real part *ε*_1_(*ω*), (b) imaginary part *ε*_1_(*ω*), (c) refractive index *n*(*ω*), (d) the extinction coefficient *k*(*ω*), (e) absorption *α*(*ω*), and (f) reflectivity *R*(*ω*) of FeIn_2_S_4_ and NiIn_2_S_4_.

Fig. 9(a and b) depict the dependence of the real and imaginary parts of the dielectric function on the photon energy in the region of 0–4 eV at pressure 0 GPa, 2 GPa and 4 GPa. The static dielectric constants (*ε*_0_) of FeIn_2_S_4_ and NiIn_2_S_4_ have been determined to be 22.40 and 54.68, respectively. High *ε*_0_ can be advantageous since it suppresses charge trapping and enhances light absorption efficiency by minimizing the rate of recombination of electron–hole pairs.^62^ A change in the transition metal from Fe to Ni results in the systematic change in the dielectric response accompanied by a red shift in the *ε*_1_ spectrum to lower energies.

The onset of the transitions is related to the direct transitions from the valence band maximum (VBM) to the conduction band minimum (CBM). These occur at energies around 0.4 and 1.6 eV for FeIn_2_S_4_ and NiIn_2_S_4_, respectively, in agreement with the band gap estimated from the band structure calculations. *ε*_1_(*ω*), is positive at low energies, changes to negative at the vicinity of 0.5–0.8 eV, and then it is positive at higher energies. The negative value of the *ε*_1_(*ω*) means that the optical characteristics in this energy range are metallic. In case of spin-polarized optical characteristics, metallic response expected for half-metallic system. There is also a tendency to increase *ε*_1_ with the increase in ionic radii of the transition metal ions. Dielectric function *ε*_1_ exhibits a slight decrease in peak intensity and a small shift to higher energy with increasing pressure 0 to 4 GPa, indicating a decreased polarizability because of compression of the lattice.

The optical absorption, denoted by the imaginary part *ε*_2_, demonstrates pronounced maxima at around 0.5 eV for FeIn_2_S_4_ and 0.4 eV for NiIn_2_S_4_. In this context, the optical absorption for NiIn_2_S_4_ is stronger, owing to the narrower band gap, as illustrated in Fig. 9(b). Fig. 9(c) illustrates the refractive index *n*(*ω*), which shows a tendency analogous to that of the real dielectric function. The static value of the refractive index *n*(0) lies between 4.9 and 7.6 for FeIn_2_S_4,_and NiIn_2_S_4_, respectively. From calculated value of *n*(*ω*), the low-energy region experiences a decrease in the refractive index *n*(and in the same way, it is observed that the band gap increases with pressure.

The extinction coefficient *k*(*ω*), based on the dielectric function, behaves similarly across the spectrum, as shown in Fig. 9(d). The absorption coefficient (*α*), which represents the energy absorbed by a photon per unit length, is connected to the extinction coefficient by the relationship *α* = 4π*k*/*λ*.^63^ According to Fig. 9(e), the absorption bands originate from electronic transitions between the two energy bands. This effect may be explained by FeIn_2_S_4_ ability to allow interband transitions, resulting in higher absorption values compared to those of NiIn_2_S_4._ Last but not least, the reflectivity *R*, which indicates the portion of incoming light that is reflected back, is plotted in Fig. 9(f). It may be observed that at zero photon energy, reflectivity increases from 0.41 in FeIn_2_S_4_ to 0.59 in NiIn_2_S_4_. Generally, calculated optical spectra show as applied pressure will cause dielectric response to be reduced at lower energies and interband transition strengths to be modified, which can be ascribed to an increase in orbital overlap, an increase in the crystal field splitting, and the corresponding broadening of the band gap.

## 4. Conclusion

Our investigation has provided broad first-principles assessment of structural, mechanical, electronic, magnetic, and thermodynamic properties for AIn_2_S_4_ (A = Fe, Ni) spinels using WIEN2k framework. Optimized lattice parameters are 10.53 Å for FeIn_2_S_4_ and 10.45 Å for NiIn_2_S_4_, while the corresponding formation enthalpies of −0.91 eV and −0.80 eV confirm that both compounds are thermodynamically stable, with Fe phase being relatively more stable. Mechanical analysis yields Poisson's ratios of 0.29 (FeIn_2_S_4_) and 0.30 (NiIn_2_S_4_) and *B*_0_/*G* ratios of 2.05 and 2.16, respectively, indicating a clear ductile character in both spinels. Electronic structure calculations show a pressure-oriented expansion of indirect bandgaps, whereby both compounds retain semiconducting electronic characteristics in the majority-spin (spin-up) channel while simultaneously exhibiting metallic conduction properties in the minority-spin (spin-down) channel. The total magnetic moment is 4.0/2.0*µ*_B_ for each compound, dominated by the Fe/Ni sublattice, consistent with robust ferrimagnetic ordering. The thermodynamic response as function of pressure and temperature, obtained *via* quasi-harmonic Debye approach implemented in GIBBS2, reveals systematic trends in entropy and Debye temperature that shed light on thermal stability and anharmonic lattice dynamics. At 0 GP pressure and 300 K temperature, the entropy is 410 J mol^−1^ K^−1^ for FeIn_2_S_4_ and 398 J mol^−1^ K^−1^ for NiIn_2_S_4_, with respect to the Debye temperatures of 378 K and 386 K. Lastly, optical properties explored for both compounds and represent the applied pressure will cause dielectric response to be reduced at lower energies. Collectively, our results underscore promise of AIn_2_S_4_ (A = Fe, Ni) spinels as candidates for future magneto-optoelectronic and spintronic devices.

## Conflicts of interest

There are no conflicts to declare.

## Data Availability

The corresponding author will provide the data generated during the study upon a reasonable request.

## References

[cit1] Xie Y., Zhang S. Y., Yin Y., Zheng N., Ali A., Younis M., Zeng Y. J. (2025). Emerging ferromagnetic materials for electrical spin injection: towards semiconductor spintronics. npj Spintronics.

[cit2] Incorvia J. A. C., Xiao T. P., Zogbi N., Naeemi A., Adelmann C., Catthoor F., Couet S. (2024). Spintronics for achieving system-level energy-efficient logic. Nat. Rev. Electr. Eng..

[cit3] Bai L., Feng W., Liu S., Šmejkal L., Mokrousov Y., Yao Y. (2024). Altermagnetism: Exploring new frontiers in magnetism and spintronics. Adv. Funct. Mater..

[cit4] Zhang L., Liu Y., Wu M., Gao G. (2025). Electric-field-and stacking-tuned antiferromagnetic FeClF bilayer: The coexistence of bipolar magnetic semiconductor and anomalous valley Hall effect. Adv. Funct. Mater..

[cit5] Han J., Cheng R., Liu L., Ohno H., Fukami S. (2023). Coherent antiferromagnetic spintronics. Nat. Mater..

[cit6] Trier F., Noël P., Kim J. V., Attané J. P., Vila L., Bibes M. (2022). Oxide spin-orbitronics: spin-charge interconversion and topological spin textures. Nat. Rev. Mater..

[cit7] Robail M., Noor N. A., Iqbal M. W., Ullah H., Mahmood A., Naeem M. A., Shin Y. H. (2022). Comprehensive study of ferromagnetic MgNd_2_X_4_ (X=S, Se) spinels for spintronic and solar cells device applications. Ceram. Int..

[cit8] Hooch A. W., Lee S., Lee H. S., Shin H., Yoo T. Y., Ko W. (2024). *et al.*, High-valence metal-driven electronic modulation for boosting oxygen evolution reaction in high-entropy spinel oxide. Adv. Funct. Mater..

[cit9] Zhang L., Liu Y., Xu Z., Gao G. (2023). Electronic phase transition, perpendicular magnetic anisotropy and high Curie temperature in Janus FeClF. 2D Mater..

[cit10] Yasir M. A., Noor N. A., Khan M. A., Niaz S., Mumtaz S., Moussa I. M., Ullah H. (2025). Spin-polarized analysis of ferromagnetism, optoelectronic and transport characteristics of HgGd_2_(S/Se)_4_ spinels: DFT calculations. Mater. Today Commun..

[cit11] Mousa A. A., Al Azar S. M., Essaoud S. S., Berarma K., Awad A., Mahmoud N. T. (2022). *et al.*, Structural, elastic, electronic, magnetic, and thermoelectric characteristics of MgEu_2_X_4_ (X=S, Se) spinel compounds: Ab initio calculations. Phys. Status Solidi B.

[cit12] Abiodun I. C., Edem M. E., Agbor O. E. (2024). Investigation of the structural and electronic properties of ternary AB_2_X_4_ based material via density functional theory for optoelectronic applications. Commun. Phys..

[cit13] Bouhemadou A., Allali D., Boudiaf K., Al Qarni B., Bin-Omran S., Khenata R., Al-Douri Y. (2019). Electronic, optical, elastic, thermoelectric and thermodynamic properties of the spinel oxides ZnRh_2_O_4_ and CdRh_2_O_4_. J. Alloys Compd..

[cit14] Alsobhi B. O., Almeshal A. (2023). Structural, elastic, thermodynamic, electronic, magnetic, thermoelectric and optical investigation of chromate spinels TCr_2_O_4_ (T=V^2+^, Mn^2+^, Fe^2+^) for optoelectronic applications. Mater. Chem. Phys..

[cit15] Haq M. A. U., Javed M., Mumtaz R., Ullah H., ur Rehman A., Wabaidur S. M. (2024). *et al.*, Mechanical and thermal response of XFe_2_O_4_ (X=Zn, Ag and Co) spinel ferrites via IR-spectroscopy and first-principles calculations. Phys. Scr..

[cit16] Ak F., Özdemir E. G., Aliabad H. A. R. (2025). Semiconducting character analysis of RhY_2_O_4_ oxide spinel via GGA, GGA+mBJ, and GGA+U approximations. Indian J. Phys..

[cit17] Özdemir E. G., Balmumcu F. I. (2024). Magnetic, pressure-dependent elastic, and band gap calculations of VCo_2_O_4_ oxide spinel via GGA, GGA+mBJ, and GGA+U. Phys. Rev. B.

[cit18] Özdemir E. G., Doğruer S. (2023). Electronic, magnetic, and pressure-induced elastic investigations of MnY_2_O_4_ oxide spinel. Eur. Phys. J. Plus..

[cit19] Özdemir E. G., Merdan Z., Aliabad H. R. (2024). Effects of applied different potentials on electronic and half-metallic characteristics and pressure-dependent elastic and thermodynamic properties of VRu_2_Br_4_ spinel. J. Magn. Magn. Mater..

[cit20] Özdemir E. G., Doğruer S. (2023). The structural, magnetic, and pressure-induced elastic predictions of ZrPd_2_O_4_ oxide spinel via GGA, GGA+mBJ, and GGA+U approximations. J. Magn. Magn. Mater..

[cit21] Raza S. A., Mustafa G. M., Ameer M. A., Noor N. A., Farooq Z., Mumtaz S., Moussa I. M. (2025). Investigation of MnSc_2_X_4_ (X=S, Se) spinels to unveil their potential for optoelectronic and thermoelectric applications. RSC Adv..

[cit22] Kartal Y. G., Ahmed W. A. A., Özdemir E. G., Doğruer S., Balmumcu F. I., Merdan Z. (2025). An alternative material obtained for spintronic applications using first-principles approximations: TiV_2_Se_4_ spinel. Theor. Chem. Acc..

[cit23] Qadoos A., Rashid M., Alkhaldi N. D., Alresheedi N. M., Boukhris I., Mahmood Q., Nasir M. N. (2025). Electronic, magnetic, optical, and thermoelectric properties of rare earth-based CaCe_2_(S/Se)_4_ spinels for spintronic and energy harvesting applications. J. Rare Earths.

[cit24] Kattan N. A., Rouf S. A., Alkhaldi H. D., Hassan M., Al-Qaisi S., Aljameel A. I. (2025). *et al.*, Study of half metallic ferromagnetism and thermoelectric properties of the spinels MgCo_2_(S/Se)_4_ for spintronic and energy harvesting. J. Inorg. Organomet. Polym. Mater..

[cit25] Jamaï I., Ziati M., Bekkioui N., Ez-Zahraouy H. (2024). Structural, electronic, optical, thermoelectric, and thermodynamic properties of XIn_2_M_4_ (X=Cd, Zn; M=S, Se, Te) spinels for solar cell and thermoelectric devices: first-principles study. Phys. Scr..

[cit26] Blaha P. , Schwarz K., Madsen G. K., Kvasnicka D. and Luitz J., WIEN2k: an Augmented Plane Wave Plus Local Orbitals Program for Calculating Crystal Properties, Vienna, Technische Universität Wien, 2001

[cit27] Petersen M., Wagner F., Hufnagel L., Scheffler M., Blaha P., Schwarz K. (2000). Improving the efficiency of FP-LAPW calculations. Comput. Phys. Commun..

[cit28] Koller D., Tran F., Blaha P. (2012). Improving the modified Becke-Johnson exchange potential. Condens. Matter.

[cit29] Otero-de-la-Roza A., Luaña V. (2011). Gibbs2: A new version of the quasi-harmonic model code. I. Robust treatment of the static data. Comput. Phys. Commun..

[cit30] Noor N. A., Hassan M., Rashid M., Alay-e-Abbas S. M., Laref A. (2018). Systematic study of elastic, electronic, optical and thermoelectric properties of cubic BiBO_3_ and BiAlO_3_ compounds at different pressure by using ab initio calculations. Mater. Res. Bull..

[cit31] Mahmood Q., Noor N. A., Jadan M., Addasi J. S., Mahmood A., Ramay S. M. (2020). First-principle investigation of ferromagnetism and thermoelectric characteristics of MgCr_2_X_4_ (X=S, Se) spinels. J. Solid State Chem..

[cit32] Tahiri A., Naji M., Talha L., Jabar A., Ahfir R., Filali M., Idiri M. (2023). First-principles calculations study of structural, elastic, electronic and optical properties of Co_2_−xVxFeGe full-Heusler alloys. J. Electron. Mater..

[cit33] Bahhar S., Tahiri A., Idiri M., Touti R., Jabar A., Naji M. (2025). Impact of pressure on quaternary Heusler alloy LiScNiGe for optoelectronic application. Mater. Sci. Semicond. Process..

[cit34] Hrida E. M., Bahhar S., Tahiri A., Naji M., Idiri M. (2024). Pressure-induced band gap shift and enhanced optical properties of quaternary Heusler TaAlCuCo: DFT study. Opt. Quantum Electron..

[cit35] Raza S. A., Mustafa G. M., Ameer M. A., Noor N. A., Farooq Z., Mumtaz S., Moussa I. M. (2025). Investigation of MnSc_2_X_4_ (X=S, Se) spinels to unveil their potential for optoelectronic and thermoelectric applications. RSC Adv..

[cit36] Kanomata T., Ido H., Kaneko T. (1973). Magnetic and crystallographic studies of MIn_2_S_4_ (M=Mn, Fe, Co and Ni). J. Phys. Soc. Jpn..

[cit37] Evans D. J., Holian B. L. (1985). The Nose–Hoover thermostat. J. Phys. Chem..

[cit38] Shannon R. D. (1976). Revised effective ionic radii and systematic studies of interatomic distances in halides and chalcogenides. Acta Crystallogr. A.

[cit39] Zhang L., Zhao Y., Liu Y., Gao G. (2023). High spin polarization, large perpendicular magnetic anisotropy and room-temperature ferromagnetism by biaxial strain and carrier doping in Janus MnSeTe and MnSTe. Nanoscale..

[cit40] Ali M. A., Naqib S. H. (2020). Recently synthesized (Ti_1_−xMox)_2_AlC (0≤x≤0.20) solid solutions: Deciphering the structural, electronic, mechanical and thermodynamic properties via ab initio simulations. RSC Adv..

[cit41] Qureshi M. W., Ali M. A., Ma X. (2021). Screen the thermomechanical and optical properties of the new ductile 314 MAX phase boride Zr_3_CdB_4_: A DFT insight. J. Alloys Compd..

[cit42] Sajjad M., Alay-e-Abbas S. M., Zhang H. X., Noor N. A., Saeed Y., Shakir I., Shaukat A. (2015). First principles study of structural, elastic, electronic and magnetic properties of Mn-doped AlY (Y=N, P, As) compounds. J. Magn. Magn. Mater..

[cit43] Noor N. A., Alay-e-Abbas S. M., Saeed Y., Abbas S. G., Shaukat A. (2013). Ab initio study of electronic structure and magnetic properties in ferromagnetic Be_1_−xMnxSe and Be_1_−xMnxTe alloys. J. Magn. Magn. Mater..

[cit44] Zhang W., Chai C., Fan Q., Song Y., Yang Y. (2020). Six novel carbon and silicon allotropes with their potential application in photovoltaic field. J. Phys.: Condens. Matter.

[cit45] Zhandun V. S. (2021). The magnetic, electronic, optical, and structural properties of the AB_2_O_4_ (A=Mn, Fe, Co; B=Al, Ga, In) spinels: Ab initio study. J. Magn. Magn. Mater..

[cit46] Maqsood S., Javed M. A., Mumtaz S., Al-Sadoon M. K. (2024). Computational study of Cd-based chalcogenide spinels CdSm_2_(S/Se)_4_ for spintronic applications. Chalcogenide Lett..

[cit47] Mahmood Q., Nazir G., Alzahrani J., Kattan N. A., Al-Qaisi S., Albalawi H. (2022). *et al.*, Room temperature ferromagnetism and thermoelectric behavior of calcium based spinel chalcogenides CaZ_2_S_4_ (Z=Ti, V, Cr, Fe) for spintronic applications. J. Phys. Chem. Solids.

[cit48] Al-Qhtani M., Mustafa G. M., Mazhar N., Bouzgarrou S., Mahmood Q., Mera A. (2021). *et al.*, Half metallic ferromagnetism and transport properties of zinc chalcogenides ZnX_2_Se_4_ (X=Ti, V, Cr) for spintronic applications. Materials.

[cit49] Al-Daraghmeh T. M., Zayed O., Mustafa G. M., Zelai T., Younas B., Albalawi H. (2024). *et al.*, Rare earth based MgPm_2_X_4_ (X=S, Se) spinel chalcogenides for spintronic and thermoelectric applications. J. Rare Earths.

[cit50] Raebiger H., Ayuela A., Nieminen R. M. (2004). Intrinsic hole localization mechanism in magnetic semiconductors. J. Phys.: Condens. Matter.

[cit51] Saeed Y., Nazir S., Shaukat A., Reshak A. H. (2010). Ab initio calculations of Co-based diluted magnetic semiconductors Cd_1_−xCoxX (X=S, Se, Te). J. Magn. Magn. Mater..

[cit52] Elahi I., Alay-e-Abbas S. M., Nazir S., Shaukat A., Tahir M. N. (2019). Evaluation of thermodynamics and p-type ferromagnetism of C, Si and Ge doped ZnX (X=S, Se and Te) semiconductors. J. Magn. Magn. Mater..

[cit53] Bahhar S., Jabar A., Tahiri A., Louzazni M., Benyoussef S., Bahmad L. (2025). *et al.*, DFT+U screening of the physical properties of XWO_3_ (X=Sr, Cd) perovskite oxides. : Condens. Matter.

[cit54] Hosen A., Dahliah D., Mohammad N. F. A., Mousa A. A., Abu-Jafar M. S. (2025). A computational study on the comparative analysis of tetragonal complex metal hydride Q_2_FeH_5_ (Q=Mg, Ca, Sr) for hydrogen storage applications. Int. J. Hydrogen Energy.

[cit55] Gurunani B., Ghosh S., Gupta D. C. (2024). Comprehensive investigation of half-Heusler alloy: Unveiling structural, electronic, magnetic, mechanical, thermodynamic, and transport properties. Intermetallics.

[cit56] Shukla V., Bhatnagar A., Verma S. K., Pandey A. P., Vishwakarma A. K., Srivastava P. (2021). *et al.*, Simultaneous improvement of kinetics and thermodynamics based on SrF_2_ and SrF_2_@Gr additives on hydrogen sorption in MgH_2_. Mater. Adv..

[cit57] Gurunani B., Gupta D. C. (2025). First-principles investigation of thermoelectric performance in KMnZ (Z=Sn, Pb) half-Heusler alloys. RSC Adv..

[cit58] Johari G. P. (2021). Entropy, enthalpy and volume of perfect crystals at limiting high pressure and the third law of thermodynamics. Thermochim. Acta.

[cit59] Dulong P. L., Petit A. T. (1819). Recherches sur quelques points importans de la theorie de la chaleur. Ann. Chem. Phys..

[cit60] Mustafa G. M., Younas B., Alkhaldi H. D., Mera A., Alqorashi A. K., Hakami J. (2024). *et al.*, First principle study of physical aspects and hydrogen storage capacity of magnesium-based double perovskite hydrides Mg_2_XH_6_ (X=Cr, Mn). Int. J. Hydrogen Energy.

[cit61] Bakr N. A., Funde A. M., Waman V. S., Kamble M. M., Hawaldar R. R., Amalnerkar D. P., Jadkar S. R. (2011). Determination of the optical parameters of a-Si:H thin films deposited by hot wire–chemical vapour deposition technique using transmission spectrum only. Pramana.

[cit62] Saadi T., Baaziz H., Ghellab T., Latelli H., Telfah A., Charifi Z. (2025). Electronic structure, mechanical and optical properties of hydrogen storage alkaline amides XNH_2_ (X=Li, Na) compounds. Int. J. Hydrogen Energy.

[cit63] Forouhi A. R., Bloomer I. (1988). Optical properties of crystalline semiconductors and dielectrics. :Condens. Matter Mater. Phys..

